# Diethylene glycol monomethyl ether: MAK Value Documentation – Translation of the German version from 2024

**DOI:** 10.34865/mb11177e11_1or

**Published:** 2026-03-30

**Authors:** Andrea Hartwig, MAK Commission

**Affiliations:** 1 Institute of Applied Biosciences, Department of Food Chemistry and Toxicology, Karlsruhe Institute of Technology (KIT) Adenauerring 20a, Building 50.41 76131 Karlsruhe Germany; 2 Permanent Senate Commission for the Investigation of Health Hazards of Chemical Compounds in the Work Area, Deutsche Forschungsgemeinschaft, Kennedyallee 40, 53175 Bonn, Germany. Further information: Permanent Senate Commission for the Investigation of Health Hazards of Chemical Compounds in the Work Area | DFG

**Keywords:** diethylene glycol monomethyl ether, testes atrophy, fertility, developmental toxicity, metabolism, MAK value, maximum workplace concentration, peak limitation, skin absorption, Luft, air

## Abstract

The German Senate Commission for the Investigation of Health Hazards of Chemical Compounds in the Work Area (MAK Commission) summarized and evaluated the data for diethylene glycol monomethyl ether [111-77-3] to derive an occupational exposure limit value (maximum concentration at the workplace, MAK value) considering all toxi­cological end points. Relevant studies were identified from a literature search and also unpublished study reports were used. Diethylene glycol monomethyl ether is not irri­tating to the skin or eyes of rabbits. At the maximum vapour concentration of 216 ml/m^3^, diethylene glycol monomethyl ether induced no effects in a 13-week inhalation study in rats. The NOAEC is therefore 216 ml/m^3^ or higher. Taking into account the longer half-life of its toxic metabolite methoxyacetic acid in humans compared to rats, a MAK value of 10 ml/m^3^ has been derived from this study. Peak Limitation Category II with an excursion factor of 8 have been assigned because of the critical systemic effect and the long half-life of methoxyacetic acid. Diethylene glycol monomethyl ether is not clastogenic or mutagenic in vitro. Studies investigating its genotoxic or carcinogenic potential in vivo are not available. However, there is no corresponding structural alert. The critical end point of diethylene glycol monomethyl ether is developmental toxicity observed in rats and rabbits independent of maternal toxicity. The malformations are thought to be caused by the metabolite methoxyacetic acid. As the margins between the derived NOAELs and the MAK value are not sufficiently large, diethylene glycol monomethyl ether has been assigned to Pregnancy Risk Group B. Based on an in vitro skin absorption study, percutaneous absorption is expected to contribute significantly to systemic toxicity and diethylene glycol monomethyl ether has been designated with an “H”. A sensitizing potential is not expected based on the available data.

**Table:** 

**MAK value (2023)**	**10 ml/m^3^ (ppm) ≙ 50 mg/m^3^**
**Peak limitation (2023)**	**Category II, excursion factor 8**
	
**Absorption through the skin (2023)**	**H**
**Sensitization**	**–**
**Carcinogenicity**	**–**
**Prenatal toxicity (2023)**	**Pregnancy Risk Group B**
**Germ cell mutagenicity**	**–**
	
**BAT value (2023)**	**15 mg methoxy acetic acid/g creatinine**
	
Synonyms	DEGME diethylene glycol methyl ether diglycol monomethyl ether 2-(2-methoxyethoxy)ethan-1-ol methyldiglycol
Chemical name (IUPAC)	2-(2-methoxyethoxy)ethanol
CAS number	111-77-3
Structural formula	HO–(CH_2_)_2_–O–(CH_2_)_2_–O–CH_3_
Molecular formula	C_5_H_12_O_3_
Molar mass	120.15 g/mol
Melting point	< –84 °C; –70 °C (ECHA 2020)
Boiling point at 1013 hPa	192–195 °C (ECHA 2020)
Density at 20 °C	1.02–1.035 g/cm^3^ (ECHA 2020)
Vapour pressure at 25 °C	0.33 hPa (ECHA 2020)
log K_OW_	–0.47 at 20 °C (ECHA 2020)
Solubility	1000 g/l water (miscible) (ECHA 2020)
pKa value	14.87 at 25 °C (calculated) (ECHA 2020)
**1 ml/m^3^ (ppm) ≙ 4.985 mg/m^3^**	**1 mg/m^3^ ≙ 0.201 ml/m^3^ (ppm)**
	
Hydrolytic stability	no data
Uses	main use: jet fuel anti-icing agent; other uses: chemical intermediate in synthesis processes, solvent in paints and floor polishes, constituent of hydraulic brake fluids, cleaning and washing agents and disinfectants (EU 2000)

Note: The substance can occur simultaneously as vapour and aerosol.

Cited unpublished toxicological studies from companies have been made available to the Commission.

## 1 Toxic Effects and Mode of Action

After oral administration in rats, more than 95% of diethylene glycol monomethyl ether is excreted with the urine within 24 hours, mainly in the form of the acid metabolite 2-(2-methoxyethoxy)acetic acid.

Diethylene glycol monomethyl ether does not cause skin or eye irritation in rabbits. No effects were observed in a 13-week inhalation study with F344 rats up to the vapour saturation concentration of 216 ml/m^3^.

The most sensitive end point identified from the results of the available studies with repeated oral exposure was a slight increase in thymus weights in male Wistar rats after gavage administration of 500 mg/kg body weight and day for 20 days. In the female rats, this effect was observed at doses of 2000 mg/kg body weight and day and above after a shorter exposure period of 12 days. The relative testis weights were reduced in Wistar rats given oral doses of 2000 mg/kg body weight for 5 days and in albino COBS CD BR rats given 3600 mg/kg body weight and day for 6 weeks.

Diethylene glycol monomethyl ether does not cause mutagenic effects in bacteria and does not induce chromosomal aberrations in V79 cells. Studies that investigated genotoxicity in vivo or carcinogenicity are not available but there is no corresponding structural alert.

There is no evidence that diethylene glycol monomethyl ether causes sensitizing effects.

Diethylene glycol monomethyl ether induced developmental toxicity and teratogenicity in rats in prenatal developmental toxicity studies with gavage administration. Reduced body weights, reduced ossification of the sternebrae and vertebrae, and visceral variations (residual thymic tissue in the neck, dilated renal pelvis) were observed in the foetuses at doses of 600 mg/kg body weight and day and above; these effects did not occur concurrently with maternal toxicity. The incidence of skeletal variations, malformations of the vertebrae and ribs, and visceral variations and malformations, particularly those affecting the cardiovascular system, increased with the dose. In a prenatal developmental toxicity study with occlusive dermal application of diethylene glycol monomethyl ether to the skin of rabbits, an increase in skeletal variations of the cervical vertebrae and delayed ossification of the hyoid bone were observed in the foetuses at doses of 250 mg/kg body weight and day and above; maternal toxicity did not occur.

## 2 Mechanism of Action

A study investigated the endocrine activity of diethylene glycol monomethyl ether at five nuclear receptors using a reporter gene assay with the endometrial adenocarcinoma cell line (Ishikawa). The IC_10_ values (median concentration needed to suppress the activity of the positive control by 10%) were about 1 × 10^–7^ M for anti-oestrogenic effects, 5 × 10^– 5^ M for anti-androgenic effects and 5 × 10^–5^ M for anti-glucocorticoid effects (data from graph). The agonistic and antagonistic effects were calculated relative to the positive control (17β-oestradiol and dihydrotestosterone). Diethylene glycol monomethyl ether did not cause anti-thyroid or anti-progesterone effects (positive controls triiodothyronine (T3) and progesterone). The anti-androgenic activity was confirmed in a reporter gene assay using HepG2 cells (Kassotis et al. 2014, 2015; no author 2015). The results indicate that diethylene glycol monomethyl ether would have weak anti-oestrogenic, anti-androgenic and anti-glucocorticoid effects. However, the method and the results are not clearly described, contradictory data for the concentration of the positive control dexamethasone are given in the methods and results sections and these data were not corrected in the corrigendum. By contrast, the results from validated models from the ToxCast database do not suggest agonistic or antagonistic effects (US EPA 2020).

## 3 Toxicokinetics and Metabolism

### 3.1 Absorption, distribution, elimination

There are no data available for inhalation exposure to diethylene glycol monomethyl ether and for the distribution of the substance.

After oral administration in rats (see Section 3.2), more than 95% of diethylene glycol monomethyl ether is excreted with the urine within 24 hours, mainly in the form of the acid metabolite 2-(2-methoxyethoxy)acetic acid. This demonstrates that the substance is completely absorbed and has a relatively short half-life (see Table 1). The study also shows that the half-life of methoxyacetic acid increases significantly at very high doses (ECHA 2020; EU 2000; Health Council of the Netherlands 2017).

**Tab. 1:** Metabolites in the urine of rats 0 to 24 hours and 24 to 48 hours after oral administration of diethylene glycol monomethyl ether in % of the administered dose (ECHA 2020)

**Substance**	**Dose [mg/kg body weight]**					
	**500**		**1000**		**2000**	
	**0–24 hours**	**24–48 hours**	**0–24 hours**	**24–48 hours**	**0–24 hours**	**24–48 hours**
MEAA MAA	93.2 1.1	1.4 0.3	89.7 0.7	1.2 0.3	86.2 0.5	1.0 0.3
DEG DEG glucuronide DEGME	2.9 1.0 3.4	0.0 0.0 0.0	2.3 0.8 3.6	0.0 0.0 0.0	2.2 0.7 4.9	0.0 0.0 0.0

DEG: diethylene glycol; DEGME: diethylene glycol monomethyl ether; MAA: methoxyacetic acid; MEAA: 2-(2-methoxyethoxy)acetic acid

In humans, the elimination half-life of methoxyacetic acid, a metabolite of diethylene glycol monomethyl ether, was 77 hours after exposure by inhalation to a 2-methoxyethanol concentration of 16 mg/m^3^ (Groeseneken et al. 1989; Hartwig 2009 a, available in German only). In male and female rats, the elimination half-life was 12 and 14 hours, respectively, after intraperitoneal administration of a 2-methoxyethanol dose of 100 mg/kg body weight (Aasmoe and Aarbakke 1997; Hartwig 2009 a). The half-life of 2-ethoxyacetic acid after oral administration is 6 times shorter in rats than in humans. Therefore, it may be assumed that also the acid metabolites of diethylene glycol monomethyl ether have longer half-lives in humans than in animals (SCOEL 2001).

Diethylene glycol monomethyl ether (purity 98%) was absorbed through human skin in vitro at a rate of 0.206 mg/cm^2^ and hour. The permeability constant was 2.06 × 10^4^ cm/hour (Dugard et al. 1984). Assuming the exposure of 2000 cm^2^ of skin for 1 hour, this would be equivalent to an absorbed amount of 412 mg.

In an in vitro study that investigated the permeability of rat skin for diethylene glycol monomethyl ether, the substance penetrated the stratum corneum at a rate of 0.051 mg/cm^2^ and hour. The permeability coefficient was 8.0 × 10^–2^ cm/hour (McDougal et al. 2000).

### 3.2 Metabolism

Four male Sprague Dawley rats per dose group were given a single gavage dose of diethylene glycol monomethyl ether (purity 99.8%) of 500, 1000 or 2000 mg/kg body weight. The urine was collected on 2 days over a 24-hour period (0–24 hours, 24–48 hours) and the samples from each animal were analysed separately. Recovery of the substance was in the range from 95.9% to 103.3%. Almost all glycol ether metabolites are excreted with the urine. Therefore, the primary objective of this study was to investigate whether the presence of methoxyacetic acid can be used as an indicator for the metabolic pathway and to determine how this metabolite contributes to the toxic potential. An analytical approach was developed that used non-radioactively labelled substances to detect metabolites in the urine. All the expected metabolites were detected with 2-(2-methoxyethoxy)acetic acid dominating at 87% to 95%. In addition, 0.8% to 1.4% of the administered dose of diethylene glycol monomethyl ether was detected as methoxyacetic acid; however, its fraction decreased with increasing dose. These results show that the main metabolic pathway involves the oxidation of the hydroxy group, with a small fraction of the substance metabolized by cleavage of the ether bond. Small amounts of unmetabolized diethylene glycol monomethyl ether, the glucuronide conjugate and diethylene glycol were detected. Table 1 lists the most important (expected) metabolites that were found in all three doses and their percentage of the dose. In addition, trace amounts of 3 other metabolites were detected. One of these was identified as a sulfate conjugate (about 0.02%), but the other two were not identified. One of these metabolites contained nitrogen. As the unknown metabolites did not make up more than about 0.1% of the dose, it was not considered necessary to identify them (ECHA 2020; EU 2000; Health Council of the Netherlands 2017). These data were obtained from an unpublished study conducted in 2017. Nearly identical data were published by Kelsey et al. (2020); these were obtained from the same laboratory and are probably from the same study.

**Fig. 1:**
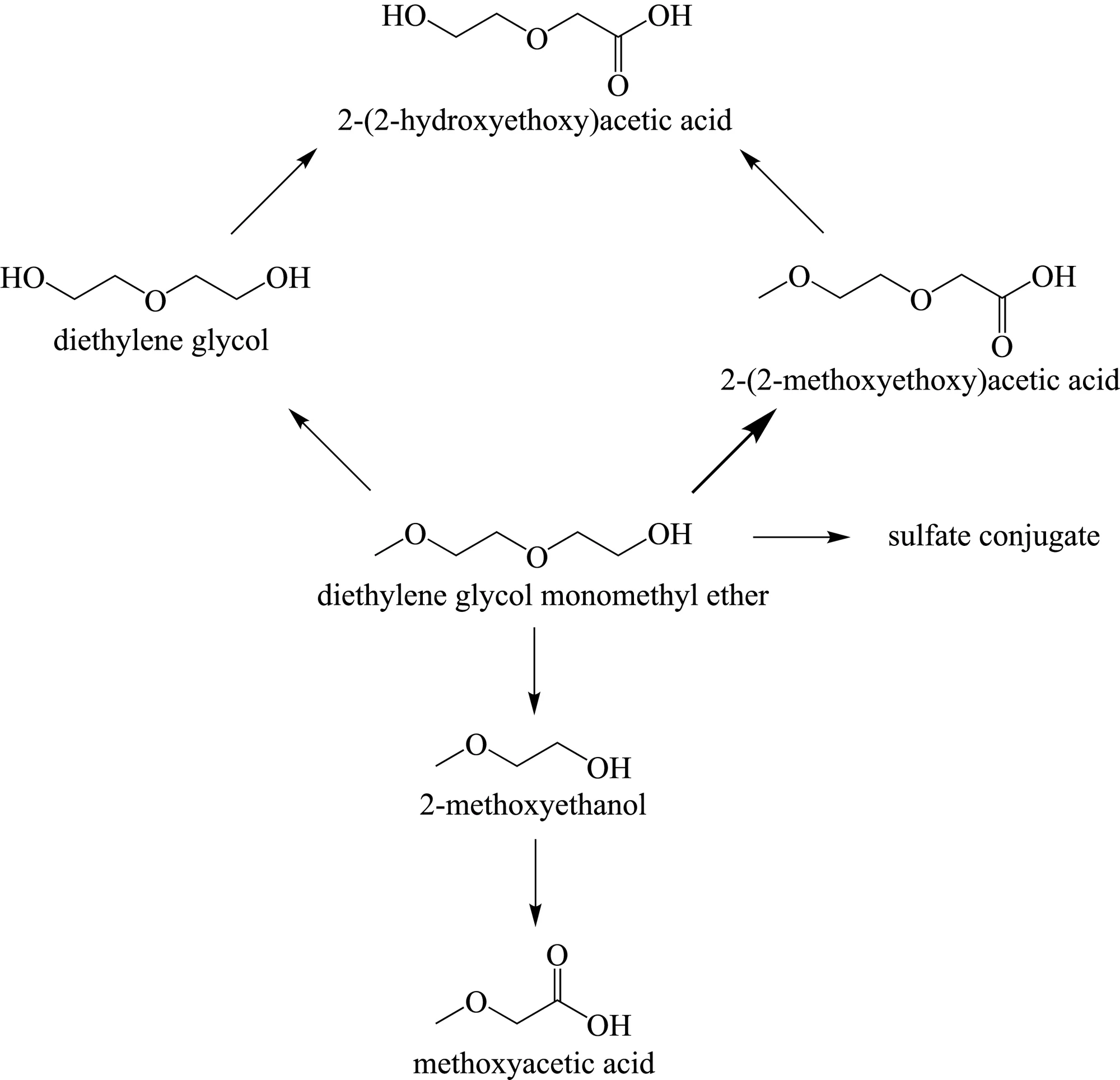
Metabolic scheme of diethylene glycol monomethyl ether according to ECHA (2019) and Kelsey et al. (2020)

Figure 1 depicts the proposed metabolic pathway of diethylene glycol monomethyl ether.

## 4 Effects in Humans

Data are available only for the end points sensitizing effects and reproductive toxicity.

### Allergenic effects

An incompletely documented maximization test carried out in 25 volunteers with a 20% formulation of diethylene glycol monomethyl ether in petrolatum yielded negative results; information about this assay is available only through a secondary source (Opdyke 1974).

### Sensitizing effects on the airways

A study of indoor background exposure investigated a possible relationship between the concentrations of volatile organic compounds (including glycol ethers) in the bedroom air and allergies and IgE sensitization in preschool-aged children. Volatile organic compounds are emitted in homes by building materials, paints, furniture, carpets, televisions, etc. The background exposure level to diethylene glycol monomethyl ether in the bedrooms of asymptomatic children was 3.91 µg/m^3^ (95% confidence interval (CI): 2.59–5.90). In the homes of 3 of the 198 children who had asthma (2 of them also rhinitis and eczema), the concentrations of diethylene glycol monomethyl ether in the indoor air were increased with statistical significance (geometric mean 7.41 µg/m^3^ (95% CI: 2.03–27.04)). However, a more in-depth investigation was not carried out to determine whether there was sensitization to diethylene glycol monomethyl ether or other glycol ethers (Choi et al. 2010). No conclusions may thus be drawn about the potential sensitizing effects of diethylene glycol monomethyl ether.

There are no data for occupational exposure.

### Reproductive and developmental toxicity

A case study from Turkey involved a 5-year-old boy with a congenital malformation and a retrocaval ureter in addition to anomalies of the cardiovascular and skeletal system. The mother was exposed to dyes while working in a textile factory during pregnancy and in the 7 years preceding her pregnancy. The authors suggested that she may have been exposed to diethylene glycol monomethyl ether (Karaman et al. 2002). However, as exposure levels were not determined and there is no evidence of exposure to the substance, the study has not been included in the evaluation.

## 5 Animal Experiments and in vitro Studies

### 5.1 Acute toxicity

#### 5.1.1 Inhalation

In a study with 5 male and 5 female Wistar rats that was published in 1984 and in a study with 3 male and 3 female rats (strain not specified) that was published in 1960, the animals were exposed to air saturated with diethylene glycol monomethyl ether for 6 or 8 hours, respectively. The exposure concentration was estimated to be 1200 mg/m^3^. No signs of toxicity or mortality were observed (ECHA 2020).

#### 5.1.2 Oral administration

A study published in 1981 investigated acute oral toxicity in fed and fasted (16 to 20 hours) male CD/BR rats. Five animals per dose group were given diethylene glycol monomethyl ether (purity > 99.5%) by gavage without a vehicle. The observation period lasted 14 days. The LD_50_ was 7128 mg/kg body weight in the fasted animals and 12 410 mg/kg body weight in the fed animals. Mortality occurred on days 1 to 10, most of the cases during the first 3 days. Both studies reported clinical symptoms of inactivity, laboured breathing, rapid respiration, anorexia, slight to moderate weakness, tremors and prostration. The animals that died had blood in the urine and/or blood in the stomach and intestines. These effects were not observed in the surviving animals that underwent necropsy at the end of the study. Haematuria was found in the animals that were exposed to the highest dose (no other details) (ECHA 2020).

In a study that was carried out in 1981 in male CD-1 mice according to the same protocol, the LD_50_ values were 7128 mg/kg body weight in the animals that were fasted for 16 to 20 hours before administration and 8188 mg/kg body weight in the fed animals. The signs of toxicity were laboured breathing, rapid respiration, anorexia, slight to moderate weakness, tremors and prostration. Mortality occurred on days 1 to 10, most of the cases during the first 3 days. The animals that died had blood in the urine and/or blood in the stomach and intestines (ECHA 2020).

These and other early oral LD_50_ studies are shown in Table 2.

**Tab. 2:** Acute toxicity studies with oral administration of diethylene glycol monomethyl ether (ECHA 2020)

**Species,** **strain,** **number per group**	**Dose [mg/kg body weight]**	**End point**	**References**
rat, CD/BR, 5 ♂	7128 12 410	LD_50_ for fasted animals LD_50_ for fed animals	study from 1981
rat, Wistar, 5 ♂, 5 ♀	about 6800–6900 (6.7 ml/kg body weight)	LD_50_	study from 1960
rat, strain not specified, 5 ♂, 5 ♀	about 6500–6600 (6.4 ml/kg body weight)	LD_50_	study from 1960
rat, Wistar, 10 ♂	9210	LD_50_	study from 1941
rat, ♂, no other data	> 5000 (no mortality) and > 10 000 (100% mortality)	LD_50_	study from 1943
mouse, CD-1, 5 ♂	7128 8188	LD_50_ for fasted animals LD_50_ for fed animals	study from 1981
rabbit, strain not specified, 8 ♂, 8 ♀	> 4000	LD_50_	study from 1961
rabbit, no other data	> 6300	LD_50_	study from 1941
guinea pig, strain not specified, 10 ♂, 10 ♀	4160	LD_50_	study from 1941
cat, breed not specified, 1 ♂, 1 ♀	> 4080	LD_50_	study from 1961

#### 5.1.3 Dermal application

In a study carried out in 1981, diethylene glycol monomethyl ether (purity > 99.5%) was applied occlusively to the skin of groups of 5 male New Zealand White rabbits in doses of 10.5, 21, 42, 84 or 168 mmol/kg body weight (about 1260, 2520, 5040, 10 080 or 20 160 mg/kg body weight) for 24 hours. The animals were then observed for 14 days. Anorexia, slight depression, cyanosis, ataxia and soft faeces were observed at low sublethal doses (no other details) and salivation, nasal discharge, iritis, lethargy, prostration and laboured breathing at higher doses (no other details). The gross-pathological findings were oedema and haemorrhage of the thymus gland, brown discoloration of the lungs and liver, greenish-brown mottled liver, discoloured kidneys with increased surface vascularity, dark red and enlarged cortex and a dark red medulla, haemorrhage of the stomach and duodenum, and dark brown fluid in the thorax and abdomen. The LD_50_ was 9404 mg/kg body weight (ECHA 2020).

Another study from 1984 was carried out with occlusive dermal exposure of groups of 5 male and 5 female New Zealand White rabbits to doses of 4, 8 or 16 ml/kg body weight. The observation period lasted 14 days. The high dose was lethal to 3 males and 3 females within 2 to 3 days. The same incidence of mortality was reached at the medium dose within 2 to 4 days; the low dose was not lethal. The surviving animals of the medium and high dose groups exhibited sluggishness, prostration and an unsteady gait, but recovered completely from the symptoms on day 2. At the low dose, only 1 male animal had an unsteady gait, which was also reversible on day 2. The main findings of the gross-pathological examination of the animals in the medium and high dose groups were discoloration of the lungs, brown mottled liver and brown fluid in the thorax and abdomen. Two males in the low dose group had dark red lungs. Erythema was observed at the application site of all exposed animals. The LD_50_ was 8.98 ml/kg body weight (9284 mg/kg body weight) (ECHA 2020).

A study carried out in 1940 determined an LD_50_ of 8000 mg/kg body weight in male and female guinea pigs. Another study carried out in 1943 with male and female guinea pigs reported a non-lethal dose of 10 000 mg/kg body weight (ECHA 2020).

### 5.2 Subacute, subchronic and chronic toxicity

#### 5.2.1 Inhalation

In a well-documented 13-week inhalation study that was carried out according to a valid method, groups of 10 male and 10 female F344 rats were exposed whole-body to diethylene glycol monomethyl ether vapour (purity > 99.5%) in concentrations of 0, 30, 100 or 216 ml/m^3^ for 6 hours a day, on 5 days a week. The highest concentration tested was the maximum vapour concentration attainable under laboratory conditions and was equivalent to more than 60% of the maximum theoretical vapour concentration at 25 °C and 1013 hPa. The body weights and organ weights did not yield any evidence of substance-induced effects, nor were these evident from the results of the haematological examinations (haematocrit, haemoglobin, erythrocytes, leukocytes, differential blood count, thrombocytes), clinical chemical analyses (urea/blood urea nitrogen (BUN), alanine aminotransferase (ALT), aspartate aminotransferase (AST), alkaline phosphatase (AP), glucose, total protein, albumin, globulin), urinalysis (bilirubin, glucose, ketones, blood, pH, protein, urobilinogen, spe­cific density) and gross-pathological and histopathological examinations (49 organs/tissues). The NOAEC (no observed adverse effect concentration) is therefore equal to or above 216 ml/m^3^ (Dow Chemical 1984 a; Miller et al. 1985).

#### 5.2.2 Oral administration

In a **12-day** dose-finding study for developmental toxicity, groups of 4 to 6 female Wistar rats that were not pregnant were given diethylene glycol monomethyl ether in gavage doses of 0, 125, 250, 500, 1000, 2000, 3000 or 4000 mg/kg body weight and day on 11 consecutive days. The body weights, feed consumption and the general state of health of the animals were determined each day. A urinalysis was carried out on day 10 within 30 minutes of treatment, using commercial test strips. The blood was analysed on day 12 and the weights of the liver, kidneys, heart, spleen, stomach, brain, adrenal glands, thymus gland, ovaries and pituitary gland were determined. The urinary pH values determined in all groups (125 mg/kg: pH 7.0–8.0; 4000 mg/kg: pH 5.0–6.0) were lower than the values of the control animals (pH 8.0–8.5). This effect was attributed to the acid metabolites of the test substance and is not an adverse effect. The only findings at 2000 mg/kg body weight and day were changes in the organ weights of the thymus and pituitary glands (no other details); however, these effects were not statistically significant. The body weight gains and feed consumption, haematocrit values and the relative pituitary gland weights were reduced with statistical significance at 3000 mg/kg body weight and day and above. The leukocyte and erythrocyte counts, haemoglobin concentrations and the relative thymus weights were reduced with statistical significance at 4000 mg/kg body weight and day. The relative kidney weights and the BUN values were slightly increased at 4000 mg/kg body weight and day. A NOAEL (no observed adverse effect level) of 1000 mg/kg body weight and day was derived based on the changes in the organ weights of the pituitary and thymus glands (Yamano et al. 1993).

In another study, diethylene glycol monomethyl ether (purity > 98%) was given daily to groups of 4 male Wistar rats in gavage doses of 0, 500, 1000 or 2000 mg/kg body weight and day for **up to 20 days**. Additional satellite groups, each made up of 4 animals, were included in the high dose group for examination after 1, 2 and 5 days. The body weights of the animals and the organ weights of the liver, kidneys, heart, thymus gland, lungs, testes and spleen were determined. In the high dose group, the body weight gains were reduced with statistical significance from day 10 of exposure and the relative organ weights of the liver, spleen, thymus gland and testes were decreased with statistical significance from day 5. After 20 days, the relative thymus weights were decreased slightly at the low dose of 500 mg/kg body weight and day and with statistical significance at 1000 mg/kg body weight and day and above (data from graph: 500 mg/kg body weight and day: about –17%, 1000 mg/kg body weight and day: about –26%, 2000 mg/kg body weight and day: about –40%). In rats, the number of lymphocytes in the thymic cortex was reduced after exposure to 2000 mg/kg body weight and day for 5 days. The NOAEL of this study is probably about 500 mg/kg body weight and day (Kawamoto et al. 1990 a). It was not reported whether additional animals were examined histopathologically.

In a **6-week** gavage study carried out in 1982, groups of 10 male CD rats (strain: CR, COBS, CD, BR albino) were given diethylene glycol monomethyl ether (purity > 99%) in doses of 0, 900, 1800 or 3600 mg/kg body weight and day on 5 days a week. Body weights were reduced in the medium dose group (7%, not statistically significant) and in the high dose group (12%, statistically significant). The animals of the high dose group also had a 13% lower feed intake; this reduction was statistically significant. A statistically significant, 16% increase in the BUN value was observed only at this dose. The changes in the organ weights did not reveal a consistent trend. The relative heart and kidney weights and the absolute spleen weights were increased with statistical significance at 3600 mg/kg body weight and day. The relative testis weights and the absolute brain and liver weights were reduced with statistical significance at this dose and testicular atrophy was observed in 6 of 10 animals. In addition, half of these animals had degenerated sperm in the epididymis and hypospermia. One animal in the same dose group had hyperkeratosis of the stomach. Protein deposits in the proximal convoluted tubules and hyaline degeneration were found in the kidneys. The latter finding was observed also in all 10 control animals and was therefore not considered exposure-related. At 1800 mg/kg body weight and day, the relative heart and testis weights were increased with statistical significance and the absolute liver weights were decreased with statistical significance. No histopathological findings that were attributable to exposure were detected in the low and medium dose groups. The only effects observed at the medium dose of 1800 mg/kg body weight and day were inconsistent increases and decreases in the relative and absolute organ weights. Exposure at the low dose of 900 mg/kg body weight and day did not induce any changes; as a result, this dose is considered the NOAEL for systemic toxicity in male rats. Female animals were not examined (The Eastman Kodak Laboratory of Industrial Medicine 1982; no other details). It is unclear why the effects on the thymus gland that were observed in the two studies above did not occur in this study.

##### 5.2.2.1 Investigation of possible liver toxicity

Groups of 4 male Wistar rats were given gavage doses of diethylene glycol monomethyl ether of 0, 500, 1000 or 2000 mg/kg body weight and day for 1 to 20 days. After administration of 2000 mg/kg body weight and day for 5 days, there was an increase in the microsomal protein content in the liver and cytochrome P450 activity was induced, but not the activities of cytochrome b5 or NADPH-cytochrome c reductase. No effects on cytosolic alcohol dehydrogenase were observed. At the high dose, the relative liver weights were decreased after administration for 5 days and the absolute liver weights were decreased after 20 days (Kawamoto et al. 1990 b). The NOAEL for this study was 1000 mg/kg body weight and day.

Under conditions identical to those of the study described above, but with exposure of rats to diethylene glycol monomethyl ether on 5 days a week for up to 4 weeks, gamma-glutamyl transpeptidase (GGT), ALT, AST or AP activities were not induced in the serum of the animals. The hepatic microsomal GGT activity was induced with statistical significance only in the high dose group exposed to 2000 mg/kg body weight and day. An examination of the liver, brain, lungs, spleen, pancreas and kidneys revealed a statistically significant, 50% increase in GGT only in the brains of the animals exposed to 1000 mg/kg body weight and day (Kawamoto et al. 1992). It was not reported whether the brains of the animals in the lower dose groups were examined. For this reason, the LOAEL (lowest observed adverse effect level) of this study was 1000 mg/kg body weight and day and a NOAEL cannot be derived.

##### 5.2.2.2 Comparison with diethylene glycol **di**methyl ether

The toxic effects induced by diethylene glycol monomethyl ether on the testes are much less severe than those induced by diethylene glycol **di**methyl ether (MAK value 1 ml/m^3^, Hartwig and MAK Commission 2023). Unlike diethylene glycol monomethyl ether, which did not induce any effects on the reproductive organs up to the highest concentration tested of 216 ml/m^3^ in a 13-week inhalation study in rats, a NOAEC of 30 ml/m^3^ was derived for adverse effects on the testes induced by diethylene glycol **di**methyl ether in a 2-week inhalation study in rats. A possible explanation is that diethylene glycol monomethyl ether and diethylene glycol **di**methyl ether are metabolized to methoxyacetic acid in different amounts. The fraction of methoxyacetic acid found in the urine (0–48 hours) of rats given oral doses of diethylene glycol monomethyl ether of 500 to 2000 mg/kg body weight and day was 0.8% to 1.4% of the applied dose and decreased with an increase in the dose (ECHA 2020; EU 2000; Health Council of the Netherlands 2017). The fraction of methoxyacetic acid is about 5 times as high after exposure to diethylene glycol **di**methyl ether (Hartwig and MAK Commission 2023).

##### 5.2.2.3 Summary

After evaluating the available studies with repeated oral exposure, a LOAEL of 500 mg/kg body weight and day was derived based on the findings after 20-day gavage administration in male Wistar rats (Kawamoto et al. 1990 a). At this dose, the thymus weights were slightly reduced, but not with statistical significance. Histopathological changes in the thymus gland in the form of a reduced number of lymphocytes in the cortex became noticeable at 2000 mg/kg body weight and day. As a result, 500 mg/kg body weight and day is regarded as the dose at which adverse effects begin to appear. The NAEL (no adverse effect level) for this study is probably about 500 mg/kg body weight and day. In the females, this effect was observed concurrently with a decrease in the relative pituitary gland weights after a shorter period of exposure of 12 days and at doses of 2000 mg/kg body weight and day and above.

#### 5.2.3 Dermal application

In a 13-week dermal study, diethylene glycol monomethyl ether was applied occlusively to the skin of groups of 6 male guinea pigs in doses of 0, 40, 200 or 1000 mg/kg body weight and day for 6 hours a day, on 5 days a week. A slight, but not statistically significant reduction in body weights was observed at the high dose. At the medium dose of 200 mg/kg body weight and day and above, the absolute and relative spleen weights of the animals were reduced with statistical significance. The lactate dehydrogenase activity in the serum was increased with statistical significance and the MCHC value (mean corpuscular haemoglobin concentration) was decreased at 1000 mg/kg body weight and day. The calcium levels in the urine were increased with statistical significance in all exposed animals. According to the authors, this is a secondary effect of the excretion of acid metabolites such as 2-(2-methoxyethoxy)acetic acid with the urine, which leads to the formation of calcium salts. As these calcium salts form, the calcium level in the urine increases while the number of calcium ions that are available for tubular reabsorption in the kidneys decreases. Other diglycolic and triglycolic acids are known to act as chelating agents for calcium and to form stable soluble calcium complexes. However, the compensatory hypercalcaemia in the blood that is known to occur with other substances was not detected. The slight accumulation of periportal fat within the hepatocytes of the liver was observed in all exposed animals; this effect was dependent on the dose (0/7, 2/6, 6/6 and 6/6, respectively, at 0, 40, 200 and 1000 mg/kg body weight and day). Focal coagulation necrosis was observed in the liver, but without dose dependency (2/7, 1/6, 4/6 and 2/6, respectively, at 0, 40, 200 and 1000 mg/kg body weight and day). This finding is difficult to evaluate, however, because no other changes that are relevant to liver toxicity occurred (Hobson et al. 1986). The Commission considers the slight accumulation of periportal fat within the hepatocytes to be difficult to evaluate because the liver weights were not increased. It is unusual to conduct a 13-week study with guinea pigs, the study used only a small number of animals (6) and only male animals. No effects occurred in the liver of rats after administration of oral doses of up to 1000 mg/kg body weight and day for 20 days and after exposure by inhalation to concentrations of up to 216 ml/m^3^ for 13 weeks. As a result, the findings are not considered relevant for the derivation of a MAK value.

### 5.3 Local effects on skin and mucous membranes

#### 5.3.1 Skin

In a study from 1984, 0.5 ml of diethylene glycol monomethyl ether (purity not specified) was applied occlusively to the shaved, intact skin of 3 male and 3 female New Zealand White rabbits for 4 hours. The animals were examined over a period of 3 days; readings were taken after 5, 24, 48 and 72 hours and scored according to Draize. The irritation scores for erythema and oedema were 0 on a scale up to 4 at all time points. Therefore, diethylene glycol monomethyl ether did not cause irritation of the skin in rabbits (ECHA 2020).

Another study was carried out in 1960 with 2 Vienna White rabbits. Diethylene glycol monomethyl ether (probably also 0.5 ml; purity not specified) was applied occlusively either to the shaved dorsal skin for 60 seconds, for 5 or 15 minutes or for 20 hours or to the skin of the ears for 20 hours. The observation period lasted 8 days. The authors of the registration dossier converted the original study data (no other details) to Draize scores, which resulted in a score of 0.16 on a scale up to 4 for erythema after exposure for 20 hours (not specified whether this was after application to the back or to the ear) and of 0 of 4 for oedema. The effects were reversible after 48 hours. The substance was assessed as not irritating to the skin of rabbits (ECHA 2020).

Diethylene glycol monomethyl ether was applied in undiluted form or as a 5%, 10%, 20% or 50% aqueous solution to the shaved skin on the right side of the body of 50 guinea pigs in total (the duration of exposure and whether the application was occlusive or non-occlusive were not specified). Hyperaemia was induced by the undiluted substance and there was slight peeling of the skin at the end of the experiment. The aqueous solutions did not cause lesions on the skin of the guinea pigs. No changes to the skin were visible after the application of 5%, 10%, 20% or 50% aqueous solutions of diethylene glycol monomethyl ether to the skin of 50 white rats and 15 rabbits for 10 days (Pastushenko et al. 1985). This study is not included in the evaluation because the method was not described in sufficient detail.

**Conclusion**: Diethylene glycol monomethyl ether does not cause irritation of the skin in rabbits.

#### 5.3.2 Eyes

In a study from 1984, 0.1 ml of diethylene glycol monomethyl ether (purity not specified) was instilled into one eye of each of 6 New Zealand White rabbits. The effects on the eyes were scored according to the Draize method over a 3-day period after 1, 4, 24, 48 and 72 hours. It was not reported whether the eyes were rinsed. The primary irritation index (24, 48, 72 hours) was 0.53; the individual scores were 0.1 of a maximum of 3 for conjunctival redness, 0.1 of a maximum of 4 for conjunctival swelling and 0 of a maximum of 4 and 2, respectively, for corneal opacity and iritis. The findings were reversible within 48 hours. Iritis was observed in half of the animals after 4 hours; this effect was, however, reversible after 24 hours. Diethylene glycol monomethyl ether caused, at most, slight to moderate irritation of the eyes in rabbits (ECHA 2020).

In another study from 1960, 0.05 ml of diethylene glycol monomethyl ether (purity not specified) was instilled into one eye of 2 Vienna White rabbits. The eyes were not rinsed and were observed for 48 hours after instillation. The primary irritation index (24, 48 hours) was 0. An initial marked reaction (no other details) was observed in the first few hours, but the findings were reversible within 24 hours. The substance was assessed as not irritating to the eyes of rabbits (ECHA 2020).

**Conclusion**: Diethylene glycol monomethyl ether does not cause irritation of the eyes in rabbits.

### 5.4 Allergenic effects

#### 5.4.1 Sensitizing effects on the skin

A maximization test with diethylene glycol monomethyl ether (purity: 99.93%) in 10 female Pirbright White guinea pigs (strain: HsdPoc:DH) yielded negative results. The undiluted test substance was used for epidermal induction. After dermal induction, severe oedema, scabs and signs of necrosis were observed around the site where Freund’s adjuvant had been injected. As marked irritation occurred on the skin after intradermal application of Freund’s adjuvant (both with the 5% test substance and without the test substance), sodium lauryl sulfate was not applied to prepare the skin for epidermal induction. No skin irritation was observed in the areas that were treated only with the test substance or a saline solution. Negative results were obtained at the challenge with the undiluted test substance (ECHA 2020).

Another study with guinea pigs is available, but it is not fully documented. In this study, 0.2 ml of diethylene glycol monomethyl ether in doses of 0.08, 0.8 or 8 mg/kg body weight (no other details) or physiological saline solution were injected subcutaneously into the outer skin of the ears of 10 guinea pigs per dose group. Epidermal application began 10 days later and continued for 7 days. Negative results were obtained at the challenge (no other details). Diethylene glycol monomethyl ether was not sensitizing (EU 2000; Pastushenko et al. 1985). This test is not included in the evaluation because the method was not described in sufficient detail.

#### 5.4.2 Sensitizing effects on the airways

There are no studies available.

### 5.5 Reproductive and developmental toxicity

#### 5.5.1 Fertility

No generation studies or mating studies with diethylene glycol monomethyl ether are available.

Studies that investigated the effects on the reproductive organs are listed in Table 3.

After whole-body exposure for 13 weeks (see also Section 5.2.1), no effects on the male and female reproductive organs (testes, epididymis, seminal vesicles, prostate glands, coagulation glands, ovaries, oviducts, uterus, cervix, vagina) were observed in F344 rats up to the high diethylene glycol monomethyl ether concentration of 216 ml/m^3^ (Dow Chemical 1984 a; Miller et al. 1985).

After 5 days, the relative testis weights were decreased in Wistar rats given gavage doses of diethylene glycol monomethyl ether of 2000 mg/kg body weight and day (see also Section 5.2.2). This effect was not yet noticeable after 20 days at 1000 mg/kg body weight and day (Kawamoto et al. 1990 a). The testes were not examined histologically.

In another study, Sprague Dawley rats were given gavage doses of diethylene glycol monomethyl ether of 613 mg/kg body weight and day for a period of up to 20 days; only one dose level was tested. The gross-pathological and histopathological examinations did not reveal any noticeable findings in the testes (Cheever et al. 1988).

In a 6-week gavage study in albino COBS CD BR rats, the relative testis weights were reduced at doses of 1800 mg/kg body weight and day and above and testicular atrophy, degenerated sperm in the epididymis and hypospermia were detected at 3600 mg/kg body weight and day (The Eastman Kodak Laboratory of Industrial Medicine 1982).

Occlusive dermal application (see also Section 5.2.3) to the skin of male Hartley guinea pigs for 13 weeks did not cause any effects on the testes and seminal vesicles at 1000 mg/kg body weight and day (Hobson et al. 1986).

The evidence shows that the male reproductive organs (testicular atrophy) are the target organs of toxicity for several glycol ethers such as methoxyethanol and ethoxyethanol. The toxic effects and the resulting impaired fertility are mediated by methoxyacetic acid and ethoxyacetic acid, respectively (ECETOC 2005).

**Tab. 3:** Studies investigating the effects of diethylene glycol monomethyl ether on the reproductive organs

**Species,** **strain,** **number per group**	**Exposure**	**Findings**	**References**
**Inhalation**			
**rat**, F344, 10 ♂, 10 ♀	**13 weeks, 6 hours/day, 5 days/week**, 0, 30, 100, 216 ml/m^3^, purity: > 99.5%	**216 ml/m^3^**: **NOAEC reproductive organs**	Dow Chemical 1984 a; Miller et al. 1985
**Oral**			
**rat**, Wistar, 4 ♂ for 1–5 days, 8 ♂ for 20 days	**1, 2, 5 days**, 0, 2000 mg/kg body weight and day, **20 days**, 0, 500, 1000, 2000 mg/kg body weight and day, gavage, vehicle: water, purity: > 98%	**1000 mg/kg body weight (20 days)**: **NOAEC reproductive organs**; **2000 mg/kg body weight**: body weights ↓ (10 days: about –10%, data from figure), relative testis weights ↓ (5 days: –16%, 20 days: –19%); no histological examination of the testes	Kawamoto et al. 1990 a
**rat**, Sprague Dawley, 5 ♂	**20 days**, 0, 5.1 mmol/kg body weight and day (613 mg/kg body weight and day), gavage, vehicle: water, purity: 99%, examination of the testes every 2 days (n = 5)	**613 mg/kg body weight**: **NOAEC reproductive organs**	Cheever et al. 1988
**rat**, Albino COBS CD BR, 10 ♂	**6 weeks, 5 days/week**, 0, 900, 1800, 3600 mg/kg body weight and day, gavage, vehicle: no data, purity: no data	**900 mg/kg body weight**: **NOAEC reproductive organs**; **1800 mg/kg body weight and above**: testes: weights ↓; **3600 mg/kg body weight**: testes: atrophy (5/10), epididymis: degenerated sperm, hypospermia	The Eastman Kodak Laboratory of Industrial Medicine 1982
**Dermal**			
**guinea pig**, Hartley, 6 ♂	**13 weeks, 6 hours/day, 5 days/week**, 0, 40, 200, 1000 mg/kg body weight and day, dermal, occlusive, purity: 99%	**1000 mg/kg body weight**: **NOAEC reproductive organs**	Hobson et al. 1986

**Summary**: Oral doses of diethylene glycol monomethyl ether caused a decrease in the relative testis weights of Wistar rats after 5 days at a dose of 2000 mg/kg body weight (Kawamoto et al. 1990 a) and of albino COBS CD BR rats after 6 weeks at doses of 1800 mg/kg body weight and day and above. Testicular atrophy, degenerated sperm in the epididymis and hypospermia were observed at 3600 mg/kg body weight and day (The Eastman Kodak Laboratory of Industrial Medicine 1982). However, no effects on the testes were detected in rats after inhalation exposure for 13 weeks to the highest concentration tested of 216 ml/m^3^ (Dow Chemical 1984 a; Miller et al. 1985).

#### 5.5.2 Developmental toxicity

The developmental toxicity studies of diethylene glycol monomethyl ether are shown in Table 4.

**Tab. 4:** Developmental toxicity studies with administration of diethylene glycol monomethyl ether

**Species,** **strain,** **number per group**	**Exposure**	**Findings**	**References**
**Oral**			
**rat**, Sprague Dawley (Crl:CD (SD)BR), 9 ♀	**GD 7–16**, dose-finding study, 0, 1000, 1495, 2235, 3345, 5175 mg/kg body weight and day, gavage, purity: no data, vehicle: water, examination GD 21	**1495 mg/kg body weight and above**: foetuses: reduced ossification of the skull; **2235 mg/kg body weight and above**: foetuses: ♀: body weights ↓, skeletal variations (vertebrae), visceral variations and malformations (cardiovascular), skeletal malformations (fused ribs, cleft sternebrae); **3345 mg/kg body weight and above**: dams: body weights ↓, feed consumption transiently reduced, number of surviving litters/pregnant dam ↓, foetuses: number of surviving foetuses/litter ↓, ♂: body weights ↓; **5175 mg/kg body weight**: dams: mortality (2/9)	Hardin et al. 1986
**rat**, Sprague Dawley (Crl:CD (SD)BR), 25 ♀	**GD 7–16**, 0, 720, 2165 mg/kg body weight and day, gavage, purity: no data, vehicle: water, examination GD 21, **similar to OECD TG 414** (2 dose groups instead of 3, doses given GD 7–16 instead of GD 5–15)	**no NOAEL for developmental toxicity**; **720 mg/kg body weight**: **NOAEL maternal toxicity**; **720 mg/kg body weight and above**: foetuses: skeletal variations/malformations (rudimentary cervical ribs, wavy/fused ribs: not evaluated separately) and visceral variations (dilated renal pelvis), reduced ossification of the appendicular skeleton (limbs, shoulder girdle and pelvic girdle) and of the skull; **2165 mg/kg body weight**: dams: body weights ↓ (GD 21), feed consumption transiently reduced, foetuses: surviving foetuses/litter ↓ (60.5% ± 31.5%, controls: 90.7% ± 8.8%), body weights ↓, reduced ossification of the vertebrae, sternebrae and ribs, skeletal malformations (rudimentary cervical ribs, wavy/fused ribs: not evaluated separately; wavy ribs: variation, fused ribs: malformation; BfR 2020), visceral malformations (cardiovascular: double aortic arch, right-sided aortic arch, right-sided ductus arteriosus, ventricular septal defect)	Hardin et al. 1986
**rat**, Wistar, 4–6 ♀	**GD 7–17**, dose-finding study, 0, 125, 250, 500, 1000, 2000, 3000, 4000 mg/kg body weight and day, gavage, purity: > 99%, vehicle: water, examination GD 20	**1000 mg/kg body weight and above**: foetuses: variations and malformations; **2000 mg/kg body weight and above**: dams: body weight gains ↓, foetuses: incidence of dead or resorbed foetuses (late stage) ↑, ♂: body weights ↓; **3000 mg/kg body weight and above**: dams: feed consumption ↓, relative pituitary gland weights ↓, haematocrit ↓, foetuses: no surviving foetuses; **4000 mg/kg body weight**: dams: relative kidney weights ↑, relative thymus weights ↓, haemoglobin ↓	Yamano et al. 1993
**rat**, Wistar, 22 ♀	**GD 7–17**, 0, 200, 600, 1800 mg/kg body weight and day, gavage, purity: > 99%, vehicle: water, examination GD 21 (14 dams) or PND 21 (8 dams) **similar to OECD TG 414 **(fewer than the minimum recommended number of 16 animals for the examination of teratogenicity, doses given GD 7–17 instead of GD 5–15)	examination GD 21: **200 mg/kg body weight**: **NOAEL developmental toxicity**; **600 mg/kg body weight**: **NOAEL maternal toxicity**; **600 mg/kg body weight and above**: foetuses: body weights ↓, visceral variations (residual thymic tissue in the neck, dilated renal pelvis), reduced ossification of sternebrae and vertebrae; **1800 mg/kg body weight**: dams: body weight gains and body weights ↓, feed consumption ↓, absolute thymus weights ↓, foetuses: number of surviving foetuses ↓, incidence of dead or resorbed foetuses (early and late stages) ↑, external malformations (anasarca, absent tail), external anomalies (back: subcutaneous haematoma), skeletal variations (vertebral clefting), skeletal malformations (absent vertebral bodies, absent digits in the fingers or toes), visceral malformations (cardiovascular: right-sided aortic arch, ventricular septal defect), reduced ossification of the cranial bones and limbs; examination PND 21: **200 mg/kg body weight**: **NOAEL perinatal toxicity**; **600 mg/kg body weight**: **NOAEL maternal toxicity**; **600 mg/kg body weight and above**: offspring: viability ↓; **1800 mg/kg body weight**: dams: prolonged gestation, offspring: number of surviving offspring ↓	Yamano et al. 1993
**mouse**, CD-1, 50 ♀	**GD 7–14**, 0, 4000 mg/kg body weight and day, gavage, purity: 99%, vehicle: water, examination PND 0–3	**4000 mg/kg body weight**: dams: mortality (5/50), offspring: number of litters with surviving offspring ↓ (5/32); number of living foetuses/litter ↓, survival PND 1–3 ↓; no examination of teratogenicity	NIOSH 1984; Schuler et al. 1984
**Dermal**			
**rabbit**, New Zealand White, 25 ♀	**GD 6–18**, 0, 50, 250, 750 mg/kg body weight and day, occlusive dermal (continuous from GD 6–18), purity: 99.6%, vehicle: water, examination GD 29, **similar to OECD TG 414**	**50 mg/kg body weight**: **NOAEL developmental and maternal toxicity**; **50 mg/kg body weight**: dams: mortality (1/25; unknown cause); **250 mg/kg body weight and above**: foetuses: delayed ossification of the hyoid bone, skeletal variations (cervical spurs); **750 mg/kg body weight**: dams: mortality (2/25; no specific cause determined at necropsy), body weight gains transiently decreased, haematological changes (erythrocyte count ↓, haematocrit ↓), foetuses: skeletal variations (mild forelimb flexure), visceral variations (dilated renal pelvis, retrocaval ureter), delayed ossification of the sternebrae; no teratogenicity	Dow Chemical 1984 b; Scortichini et al. 1986

GD: gestation day; PND: postnatal day; TG: test guideline

In a dose-finding study in Sprague Dawley rats (Crl:CD (SD)BR) with gavage administration of diethylene glycol monomethyl ether from gestation days 7 to 16, reduced ossification of the skull was observed in the foetuses at doses of 1495 mg/kg body weight and day and above without concurrent maternal toxicity. In the main study, skeletal variations of the ribs, reduced ossification of the limbs, the shoulder girdle and pelvic girdle, and visceral variations (dilated renal pelvis) were detected in the foetuses at the low dose of 720 mg/kg body weight and day and above without concurrent maternal toxicity. The incidence of skeletal variations and cardiovascular malformations increased with the dose (Hardin et al. 1986). A NOAEL for developmental toxicity could not be derived from the findings of this study.

Another dose-finding study with gavage administration in Wistar rats from gestation days 7 to 17 found an increased incidence of variations and malformations at diethylene glycol monomethyl ether doses of 1000 mg/kg body weight and day and above without concurrent maternal toxicity. In the main study, no maternal toxicity, but reduced body weights, visceral variations (residual thymic tissue in the neck, dilated renal pelvis) and reduced ossification of the sternebrae and vertebrae were observed in the foetuses at doses of 600 mg/kg body weight and day and above. Malformations occurred with an increase in the dose. The NOAEL for developmental toxicity was 200 mg/kg body weight and day, the NOAEL for maternal toxicity 600 mg/kg body weight and day (Yamano et al. 1993).

Diethylene glycol monomethyl ether given to CD-1 mice by gavage from gestation days 7 to 14 led to an increase in mortality in the dams and reduced the number of living offspring at the only dose administered of 4000 mg/kg body weight and day (NIOSH 1984; Schuler et al. 1984).

After occlusive dermal application of diethylene glycol monomethyl ether to New Zealand White rabbits from gestation days 6 to 18, an increase in skeletal variations (cervical spurs) and delayed ossification of the hyoid bone were observed in the foetuses at doses of 250 mg/kg body weight and day and above without concurrent maternal toxicity. Further skeletal variations (mild forelimb flexure), delayed ossification of the sternebrae and visceral variations (dilated renal pelvis, retrocaval ureter) were observed at 750 mg/kg body weight and day. At this dose, the body weight gains of the dams were reduced and haematological changes were detected. Teratogenic effects were not found up to the high dose of 750 mg/kg body weight and day. The NOAEL for maternal toxicity and developmental toxicity was 50 mg/kg body weight and day (Dow Chemical 1984 b; Scortichini et al. 1986).

In a study in Alpk/Ap rats with subcutaneous administration of diethylene glycol monomethyl ether, the survival of the offspring was slightly reduced at 1000 µl/kg body weight and day (equivalent to about 1020 mg/kg body weight and day) (Doe 1984). The scope of the study was limited and the study is therefore not suitable for the evaluation.

Several glycol ethers, such as methoxyethanol and ethoxyethanol, are known to cause teratogenic effects. Malformations are induced in the skeletal and cardiovascular systems, the central nervous system and the urogenital tract. These effects are mediated by methoxyacetic acid and ethoxyacetic acid, respectively (ECETOC 2005).

Methoxyacetic acid given in oral doses to rats caused cardiovascular (mainly a double aortic arch or abnormal positioning of the aortic arch) and skeletal malformations such as fused ribs, absent digits in the fingers and toes and an absent tail (Nelson et al. 1989; Ritter et al. 1985). Diethylene glycol monomethyl ether induced cardiovascular malformations in rats such as a double aortic arch, a right-sided aortic arch, a right-sided or absent ductus arteriosus and ventricular septal defects. Other skeletal malformations were observed including fused ribs, absent vertebral bodies, absent digits in the fingers and toes (Hardin et al. 1986; Yamano et al. 1993), anasarca and an absent tail (Yamano et al. 1993). The kinds of malformations induced by diethylene glycol monomethyl ether are thus qualitatively similar to those induced by methoxyacetic acid (ECETOC 2005).

##### In vitro

To study chondrogenesis, isolated forelimb buds from fertilized chicken eggs were incubated with diethylene glycol monomethyl ether for up to 14 days. The substance reduced the proteoglycan content and decreased cell proliferation at the highest concentration tested of 100 μl/ml. A concurrent increase in apoptotic cells was not observed. By contrast, methoxyacetic acid reduced the proteoglycan content, decreased cell proliferation and increased the number of apoptotic cells in a concentration-dependent manner. The authors concluded that methoxyacetic acid impairs chondrogenesis via apoptosis (Scofield et al. 2006).

### 5.6 Genotoxicity

#### 5.6.1 In vitro

A mutagenicity test published in 2017 was carried out according to OECD Test Guideline 471 in the Salmon­ella typhimurium strains TA98, TA100, TA1535 and TA1537 and in Escherichia coli WP2uvrA. Diethylene glycol monomethyl ether concentrations (purity not specified, dissolved in dimethyl sulfoxide (DMSO)) of 0, 1.5, 5, 15, 50, 150, 500, 1500 or 5000 µg/plate were tested in the plate incorporation assay, while concentrations of 0, 15, 50, 150, 500, 1500 or 5000 µg/plate were tested using the pre-incubation method. The tests were carried out with and without metabolic activation. Diethylene glycol monomethyl ether did not cause mutagenic effects in any of the test batches. Neither cytotoxicity nor precipitate formation was observed up to the highest concentration (ECHA 2020).

Another study published in 1989 conformed overall with the procedure established by OECD Test Guideline 471, but did not include a test with Escherichia coli. The mutagenic potential of diethylene glycol monomethyl ether (purity 99.9%) was investigated in the Salmonella typhimurium strains TA98, TA100, TA1535 and TA1537. Diethylene glycol monomethyl ether concentrations (in DMSO) in a range from 20 to 5000 µg/plate (no other details) were tested in the standard plate test and in the plate incorporation test both with and without metabolic activation. Diethylene glycol monomethyl ether did not cause mutagenic effects in any of the test batches. Neither cytotoxicity nor precipitate formation was observed up to the highest concentration tested (ECHA 2020).

The NTP carried out a mutagenicity assay with the Salmonella typhimurium strains TA97, TA98, TA100 and TA1535 using the pre-incubation method both in the absence and presence of a metabolic activation system. The incidence of mutations was not increased at diethylene glycol monomethyl ether concentrations of 0, 100, 333, 1000, 3333 or 10 000 µg/plate. Neither cytotoxicity nor precipitate formation was observed up to the highest concentration tested (NTP 2018).

Several international assessments include an unpublished study carried out in 1997 by the company Höchst AG (the original report is no longer available). In the study, a chromosomal aberration test conducted with diethylene glycol monomethyl ether in V79 cells yielded negative results. The study was carried out according to OECD Test Guideline 473 and under GLP conditions. In two separate experiments, diethylene glycol monomethyl ether was tested in concentrations of up to 1201.7 µg/ml (= 10 mM) both in the presence and in the absence of a metabolic activation system. In both experiments, the mitotic index was reduced after 28 hours, but not after 20 hours. Diethylene glycol monomethyl ether did not induce chromosomal aberrations. The functioning of the test system was verified by the positive controls ethyl methanesulfonate and cyclophosphamide (EU 2000; SCOEL 2001).

**Summary**: Diethylene glycol monomethyl ether did not cause mutagenic effects in bacteria in three experiments and did not induce chromosomal aberrations in a study in V79 cells. These results were obtained in valid studies that were carried out in conformity with the test guidelines.

#### 5.6.2 In vivo

There are no data available.

### 5.7 Carcinogenicity

There are no data available.

## 6 Manifesto (MAK value/classification)

The critical effect is developmental toxicity.

**MAK value. **There are no data in humans available. In a 13-week inhalation study in F344 rats, substance-induced effects were not observed after exposure to diethylene glycol monomethyl ether at the maximum vapour concentration of 216 ml/m^3^ for 6 hours a day, on 5 days a week. Therefore, the NOAEC is equal to or above 216 ml/m^3^ (Miller et al. 1985).

After evaluating the studies that investigated repeated oral exposure, a LOAEL of 500 mg/kg body weight and day was derived for 20-day gavage administration in male Wistar rats. A slight, 17% reduction in the relative thymus weight was observed at this dose (Kawamoto et al. 1990 a). The NAEL for this study is probably close to 500 mg/kg body weight and day. In female animals, this effect was observed in a developmental toxicity study after a shorter period of exposure of 12 days and at doses of 2000 mg/kg body weight and day and above (Yamano et al. 1993). The LOAEL of 500 mg/kg body weight and day corresponds to a concentration of 347 ml/m^3^ (1736 mg/m^3^) after toxicokinetic conversion (respiratory volume of 0.8 l/min/kg body weight, daily exposure for 6 hours, experimentally determined oral absorption of 100% and assumed inhalation absorption of 100%). The concentration calculated from the LOAEL for oral exposure is close to the NOAEC for inhalation exposure of 216 ml/m^3^ that was obtained from the findings of the 13-week study. A MAK value that is derived from this study is regarded as the worst case because of the bolus effect associated with the oral administration of the substance by gavage. The MAK value has therefore been derived based on the findings of the inhalation study, which is more relevant for exposure at the workplace.

After extrapolating the data to chronic exposure conditions (1:2), extrapolating the findings from an animal study (1:2) and taking into consideration the increased respiratory volume at the workplace (1:2 because it is a systemic effect), the NOAEC for inhalation exposure of 216 ml/m^3^ corresponds to a concentration of 27 ml/m^3^. A MAK value of 20 ml/m^3^ would then be obtained by applying the preferred value approach. In the following, the longer half-life of the toxic metabolite methoxyacetic acid in the blood of humans (77 hours) relative to its half-life in the blood of rats (12 hours) (see Section 3.1; Aasmoe and Aarbakke 1997; Groeseneken et al. 1989; Hartwig 2009 a) is taken into consideration as an additional parameter.

According to the allometric scaling principle, rats generally metabolize and excrete substances four times faster per kg body weight than humans. Therefore, the equipotent dose for humans is only 1/4 the dose given to rats. This is expressed by a half-life in humans that is four times longer than that in rats. Even though a rat at rest absorbs four times more substance per kg body weight by inhalation than a human at rest, this is compensated for by metabolic and excretory processes in rats that are four times faster than those in humans. Therefore, if exposed to the same concentration in air at the same level of absorption, the body burden per kg body weight will be the same for humans at rest and for rats at rest (see Hartwig and MAK Commission 2017). As a result, the differences in the half-lives in rats and humans are generally compensated for by applying a factor of 4. However, the toxic metabolite methoxyacetic acid has a half-life of 77 hours in the blood of humans, which is considerably longer than its half-life of 12 hours in the blood of rats. This results in a half-life ratio of 6.4. The corrected half-life ratio is applied to convert the concentration of 27 ml/m^3^ to a concentration of 17 ml/m^3^ (27 ml/m^3^ × 4/6.4). A MAK value of 10 ml/m^3^ (50 mg/m^3^) is set using the preferred value approach.

This value also approximately reflects the ratio of toxicity to diethylene glycol **di**methyl ether (see Section 5.2.2), which produces about five times the amount of the critical metabolite methoxyacetic acid that is responsible for the toxic effects on the testes. The MAK value for diethylene glycol **di**methyl ether has been set at 1 ml/m^3^ (5.6 mg/m^3^) in analogy to that for 2-methoxyethanol (Hartwig 2009 a) and its metabolite methoxyacetic acid (Hartwig 2009 b, available in German only) because data for longer periods of exposure are not available (Hartwig and MAK Commission 2023).

**Peak limitation. **The substance has been classified in Peak Limitation Category II because the critical effect is a systemic effect. As the critical metabolite methoxyacetic acid has a long half-life (see above and Section 3.1), peak exposure has been limited by an excursion factor of 8.

**Prenatal toxicity. **In prenatal developmental toxicity studies, diethylene glycol monomethyl ether caused developmental toxicity, and particularly teratogenicity, after gavage administration in rats (Hardin et al. 1986; Yamano et al. 1993). At doses of 600 mg/kg body weight and day and above, the foetuses had reduced body weights, reduced ossification of the sternebrae and vertebrae in addition to visceral variations (residual thymic tissue in the neck, dilated renal pelvis) without concurrent maternal toxicity (Yamano et al. 1993). The incidence of skeletal variations, malformations of the vertebrae and ribs, and visceral variations and malformations, particularly of the cardiovascular system, increased with the dose (Hardin et al. 1986; Yamano et al. 1993). An oral NOAEL for developmental toxicity of 200 mg/kg body weight and day and an oral NOAEL for maternal toxicity of 600 mg/kg body weight and day were derived for rats (Yamano et al. 1993). In a prenatal developmental toxicity study with occlusive dermal application of diethylene glycol monomethyl ether to rabbits, the foetuses exhibited an increased incidence of skeletal variations in the cervical vertebrae and delayed ossification of the hyoid bone at doses of 250 mg/kg body weight and day and above without concurrent maternal toxicity. Malformations were not observed up to the highest dose tested of 750 mg/kg body weight and day. The dermal NOAEL for developmental toxicity in rabbits was therefore 50 mg/kg body weight and day (Dow Chemical 1984 b; Scortichini et al. 1986). The kinds of malformations that were induced by diethylene glycol monomethyl ether are similar to those induced by methoxyacetic acid. The metabolite methoxyacetic acid is therefore assumed to be responsible for these effects (ECETOC 2005).

The following toxicokinetic data are taken into consideration for the extrapolation of the NOAELs for prenatal develop­mental toxicity of 200 mg/kg body weight and day (oral, rat) and 50 mg/kg body weight and day (dermal, rabbit) to a concentration in workplace air: the corresponding species-specific correction values for the rat and rabbit (1:4 and 1:2.4, respectively), the experimentally determined oral and assumed dermal absorption (100%), the body weight (70 kg) and the respiratory volume (10 m^3^) of the person and the assumed 100% absorption by inhalation. The concentrations calculated from this are 350 mg/m^3^ and 146 mg/m^3^, respectively. As these concentrations are only 7 and 3 times the MAK value of 10 ml/m^3^ (50 mg/m^3^), respectively, the margins between these values and the MAK value are not sufficiently large to justify classification in Pregnancy Risk Group C.

Diethylene glycol monomethyl ether causes developmental toxicity without inducing maternal toxicity. Developmental toxicity is therefore the main effect. For this reason, diethylene glycol monomethyl ether has been classified in Pregnancy Risk Group B.

The metabolites 2-methoxyethanol (Hartwig 2009 a) and methoxyacetic acid (Hartwig 2009 b) are likewise classified in Pregnancy Risk Group B.

**Carcinogenicity and germ cell mutagenicity. **Diethylene glycol monomethyl ether did not cause mutagenic effects in bacteria in three studies and did not induce chromosomal aberrations in V79 cells in another study; these were all valid studies that were carried out in conformity with the test guidelines. There are no studies of genotoxicity in vivo or carcinogenicity. The structure of the substance does not give reason to expect such effects. For this reason, diethylene glycol monomethyl ether has not been classified in one of the categories for carcinogens or germ cell mutagens.

**Absorption through the skin. **On the basis of the findings of an in vitro study (Section 3.1), the maximum amount dermally absorbed by humans has been estimated to be 412 mg after exposure to a saturated aqueous solution under standard conditions (2000 cm^2^ of skin, exposure for 1 hour). After extrapolation to humans, the systemic NOAEC was 17 ml/m^3^ (85 mg/m^3^) (see Section “MAK value”). At a respiratory volume of 10 m^3^ and inhalation absorption of 100%, this is equivalent to a systemically tolerable amount of 850 mg. The amount absorbed through the skin is therefore more than 25% of the systemically tolerable amount and the substance has been designated with an “H” (for substances which can be absorbed through the skin in toxicologically relevant amounts).

**Sensitization. **There is no evidence of sensitization in humans or positive findings from animal studies. Therefore, diethylene glycol monomethyl ether has not been designated with “Sh” (for substances which cause sensitization of the skin). There are no data for sensitizing effects on the respiratory tract. As a result, the substance has not been designated with “Sa” (for substances which cause sensitization of the airways).
